# Inhibition of infection spread by co-transmitted defective interfering particles

**DOI:** 10.1371/journal.pone.0184029

**Published:** 2017-09-15

**Authors:** Ashley Baltes, Fulya Akpinar, Bahar Inankur, John Yin

**Affiliations:** 1 Department of Chemical and Biological Engineering, University of Wisconsin-Madison, Madison, Wisconsin, United States of America; 2 Systems Biology Theme, Wisconsin Institute for Discovery, University of Wisconsin-Madison, Madison, Wisconsin, United States of America; Deutsches Primatenzentrum GmbH - Leibniz-Institut fur Primatenforschung, GERMANY

## Abstract

Although virus release from host cells and tissues propels the spread of many infectious diseases, most virus particles are not infectious; many are defective, lacking essential genetic information needed for replication. When defective and viable particles enter the same cell, the defective particles can multiply while interfering with viable particle production. Defective interfering particles (DIPs) occur in nature, but their role in disease pathogenesis and spread is not known. Here, we engineered an RNA virus and its DIPs to express different fluorescent reporters, and we observed how DIPs impact viral gene expression and infection spread. Across thousands of host cells, co-infected with infectious virus and DIPs, gene expression was highly variable, but average levels of viral reporter expression fell at higher DIP doses. In cell populations spatial patterns of infection spread provided the first direct evidence for the co-transmission of DIPs with infectious virus. Patterns of spread were highly sensitive to the behavior of initial or early co-infected cells, with slower overall spread stemming from higher early DIP doses. Under such conditions striking patterns of patchy gene expression reflected localized regions of DIP or virus enrichment. From a broader perspective, these results suggest DIPs contribute to the ecological and evolutionary persistence of viruses in nature.

## Introduction

When a virus infects a host cell, it can produce many thousands of virus particles, but most are non-infectious [1], with the ratio of total particles-to-infectious units spanning from 10-to-1 to 100,000-to-1 for diverse RNA and DNA viruses [2]. Defective interfering particles (DIPs) were discovered more than 70 years ago by von Magnus, who found that serial passage of allantoic fluid containing influenza A virus produced a large increase in material that, like virus, agglutinated red blood cells, but failed to cause infection [3, 4]. Since then, DIPs have been found in laboratory cultures of most classes of RNA and DNA viruses [5–7]. They have been isolated from patients infected with hepatitis B [8], hepatitis C [9, 10], influenza A [11], and dengue virus [12, 13]. They have also been isolated from birds infected with West Nile virus [14]. These studies provide the most compelling evidence for DIPs in nature.

**Fig 1:**
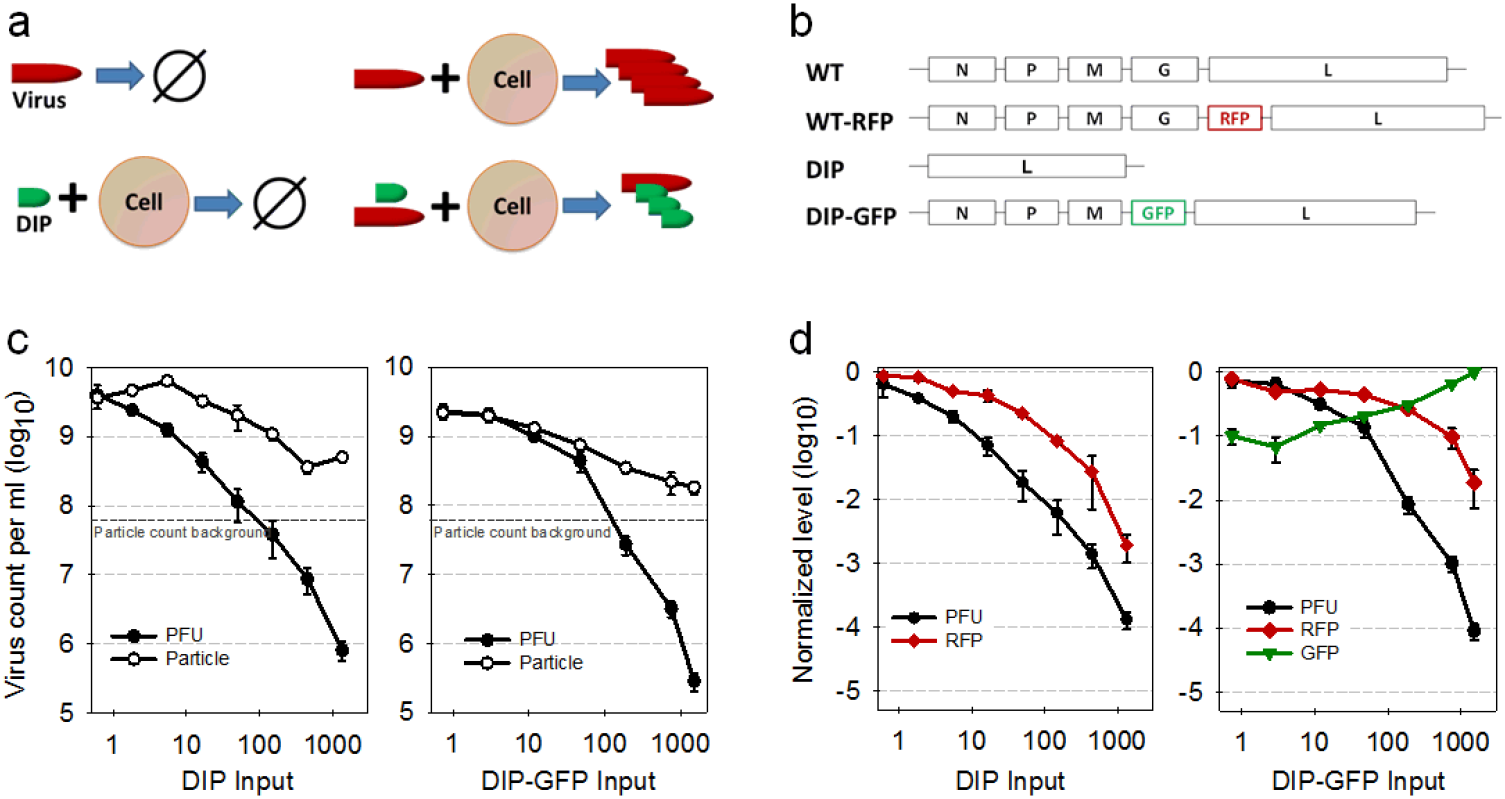
Structure and function of natural and engineered viruses. (a) Replication potential of natural infectious virus and defective interfering particles (DIPs). An infectious virus alone cannot replicate, but after it infects a permissive cell, viral progeny are produced. A DIP alone can enter a cell, but it cannot replicate. However, when an infectious virus and DIP infect the same cell, DIPs can replicate at the expense of infectious virus. Here figures highlight qualitative relationships between inputs and outputs. (b) Structure of natural and engineered virus genomes. (c) Production of particles, total and infectious (PFU), depends on level of natural (DIP) or engineered (DIP-GFP) input to co-infected cells. (d) Expression of virus or DIP reporter depends on level of natural (DIP) or engineered (DIP-GFP) inputs to co-infected cells. Values are normalized to a no-DIP control (PFU, RFP) or highest DIP input (GFP).

DIPs also arise during cell culture, with deletions occurring in one or multiple genes that are essential for growth [4, 13, 15]. Owing to these deletions, DIPs cannot replicate alone; but during co-infection with infectious virus, DIPs compete for missing viral proteins to complete their replication. Consequentially, DIPs interfere with infectious virus production (Fig 1A). Early DIP studies focused on interference during the replication stage of infection and found that DIPs reduce secondary transcription and translation in VSV and influenza virus infections [16–18]. Differences in promoter strength and genome length may provide a replicative advantage to DIPs over infectious virus [19–22]. Moreover, DIP genomes may compete with viral genomes for binding to viral structural proteins and thereby interfere with the assembly of infectious virus particles [23, 24].

Although *in vitro* and single-round infection studies have elucidated molecular mechanisms of DIP interference, interactions in nature between DIPs and their viruses may span multiple rounds of infection as they amplify within the cells and spread among tissues of their hosts. Little is known about how populations of DIPs and virus particles interact over space and time. Mathematical and computational modeling of co-infection and spread have suggested diverse possibilities [25–31]. For example, levels of virus and DIP production from co-infected cells can be highly sensitive to their input ratios (multiplicities of infection, MOI) [26], and such sensitivity to conditions can amplify during multi-cycle propagation [27, 30]. Effects of defensive cytokines, such as interferon, have also been accounted for by discrete models of DIP-virus co-infection spread [28]. Engineered reporter viruses have enabled measurement of viral gene expression during infection of susceptible host cells and multi-cycle propagation of infections [32–35]. In addition, the expression of a virus reporter was delayed and reduced by co-infection with DIPs of vesicular stomatitis virus (VSV) in a dose-dependent manner [36]. Moreover, the dynamics of infection spread across a population of healthy susceptible host cells was sensitive to DIPs in the first infected cell of the population [37]. Specifically, the expansion of virus plaques depended on the level of DIP exposure by the first infected cell, and at higher DIP input doses plaques exhibited spatially “patchy” viral reporter expression. It is not known, however, what role the timing, level, and spatial distribution of DIP replication play in the spread of infection.

Here we constructed a DIP strain of VSV encoding a green fluorescent protein (DIP-GFP), shown in Fig 1B, where DIP production depends on viral glycoprotein(G) complementation by a co-infecting infectious virus or host cell engineered to express G. This DIP-GFP strain completes all steps of the wild-type virus life cycle except packaging and particle release; notably, cells infected with only DIP-GFP make quantifiable fluorescence. We utilized this new GFP-reporter DIP and a reporter parent virus that expresses red fluorescent protein (RFP) to measure the expression of genes and the production of particles by virus-infected cells subjected to different DIP-GFP doses. Then we used the co-infected cells to initiate spreading infections in susceptible cell monolayers and tracked the distribution of both virus types in space and time by the expression of their corresponding reporter proteins. We found that DIPs and virus can co-exist, compete or dominate during infection spread, reflecting facets of their interactions that may occur in nature.

## Results

### Reporter DIP interferes with virus growth

The RFP-expressing infectious virus was previously found to grow like wild-type virus, where RFP expression correlated with the production of virus particles [34, 36]. To test the reporter DIP we compared it with natural (non-reporter) DIP in its effects on co-infection with reporter virus. When reporter virus (MOI 30) was used with different levels of natural or reporter DIP to co-infect BHK cells, the resulting virus titers were similar. Specifically, they dropped up to 10,000-fold at the highest DIP input levels (Fig 1C, left and right panels, respectively). Despite the dramatic drop in the production of infectious virus associated with high input DIP co-infections, there was only about a ten-fold drop in the corresponding production of total particles, resulting in similar average particle-to-PFU ratios of about 1000 for both natural and reporter DIPs (Fig 1C). For natural DIP co-infections at low levels of DIPs, total particle counts initially increased as DIP input levels increased, then they dropped as DIP input levels further increased and finally rebounded at the highest DIP input level. By contrast, total particle counts dropped monotonically as reporter DIP was increased from the lowest to the highest input levels.

Natural and reporter DIPs also exhibited comparable dose-dependent inhibitory effects on the expression of reporter protein (RFP) from infectious virus over the broad range of DIP input levels (Fig 1D, red curves). Natural DIPs caused RFP intensities to drop 100-to-1000 fold over the range in input DIPs, while reporter DIPs caused more modest drops in RFP intensity of 10-to-100 fold, likely reflecting a lower capacity for interference by the reporter DIPs relative to the natural DIPs. Further, the reporter DIP produced detectable GFP in a dose-dependent manner, increasing about ten-fold between the lowest and highest input levels. Thus, the dual reporters provided detectable measures of both viral and DIP gene expression from the same co-infected cells.

### Dual-color reporters show trade-offs between infectious and defective viral gene expression

**Fig 2:**
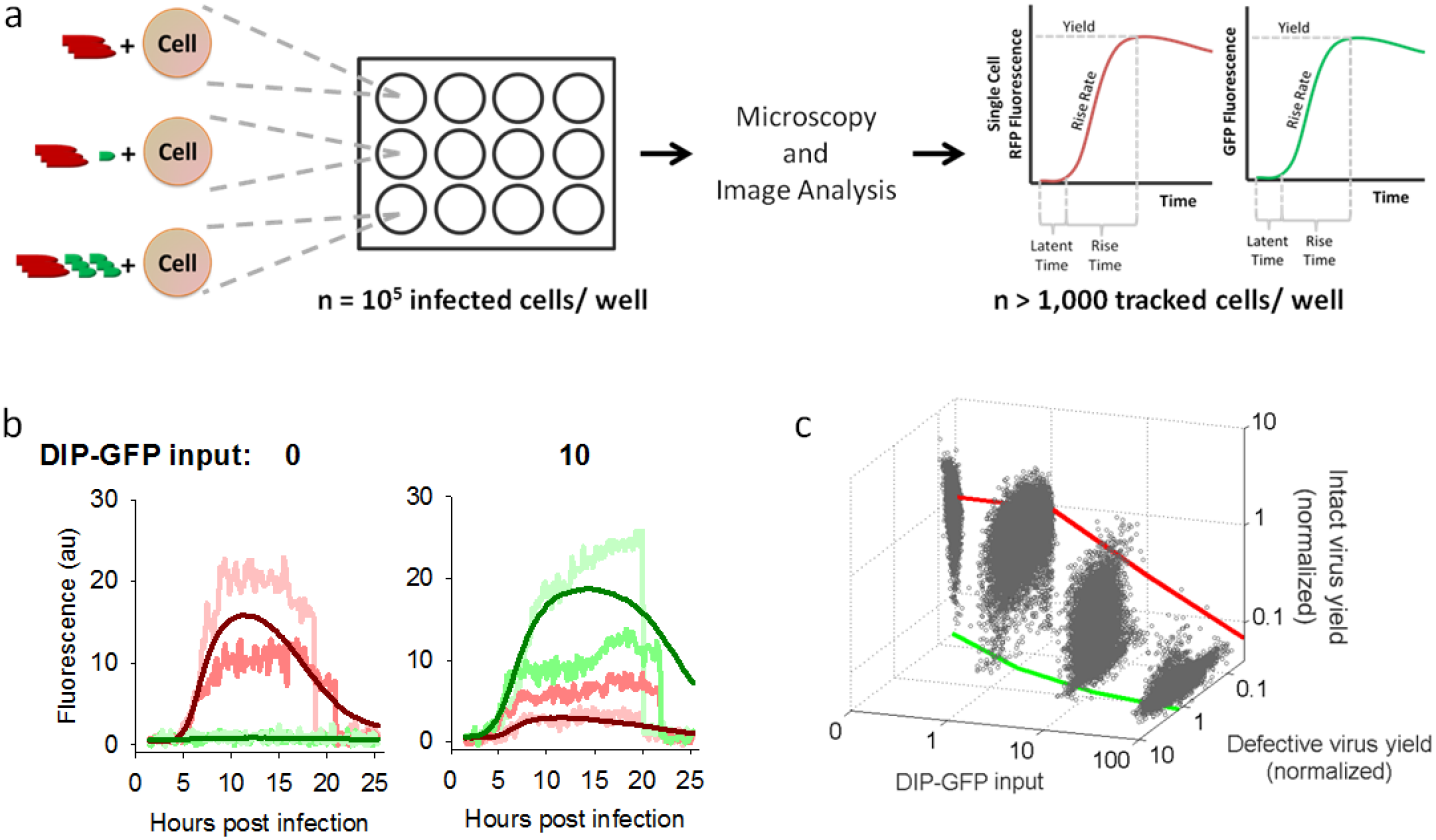
Single-cell measures of reporter expression show trade-offs between infectious and defective virus. (a) Experimental setup. Cells in all conditions were infected with a constant input of infectious virus (30 particles/cell) and varying amounts of DIP-GFP(0-to-84 particles/cell). Individual cells were imaged by time-lapse fluorescence microscopy, as detailed in Methods. For each tracked cell kinetic parameters were estimated for each RFP and GFP expression profile. (b) Example single cell kinetics. RFP and GFP kinetics are shown for two representative cells for DIP input levels 0 and 10. The average RFP and GFP expressions are also shown (dark red and green lines). (c) Anticorrelation between infectious and defective virus yields in single cells. Each gray point is an individual cell, and the green and red lines represent average defective and infectious virus yields respectively.

Although co-infection with a few DIP particles can be sufficient to prevent wild-type virus production [38], recent experiments found virus production in about 80 percent of cells co-infected with ten DIP particles per cell [36]. Here we monitored both viral and DIP expression kinetics in thousands of single cells co-infected with RFP-expressing virus (MOI 30) and varying levels of DIP-GFP (Fig 2A). The intracellular expression kinetics were visualized over time via time-lapse microscopy at the single-cell level (Fig 2B). Based on the maximum fluorescent protein expression, we determined the yield for both the infectious and defective viruses. As the DIP-GFP input level increased, the average defective virus protein yield from individual cells increased while the average infectious virus yield decreased (Fig 2C). Although there was broad heterogeneity of individual cell behaviors for each condition, trends in average yields were quite clear. The extreme cell-to-cell heterogeneity is in line with previous observations where the cell cycle state, the local environment of the cell, and stochastic gene expression can impact viral protein and particle yields [35, 36, 39, 40]. In general, there was a significant trade-off at the single cell level between reporter expression levels for infectious and defective virus co-infections (Pearson product-moment correlation coefficient is -0.49 for more than 48,000 cells).

In addition to reducing the final yield of viral reporter, DIP-GFP dose also altered its expression kinetics, with higher doses correlating with lower rise rates (S1 Fig, RFP curves). As expected, higher DIP-GFP doses also favored the kinetics of DIP reporter, reducing latent times and increasing both rise times and rise rates (S1 Fig, GFP curves).

### DIPs can propagate during spatial spread of infections

**Fig 3:**
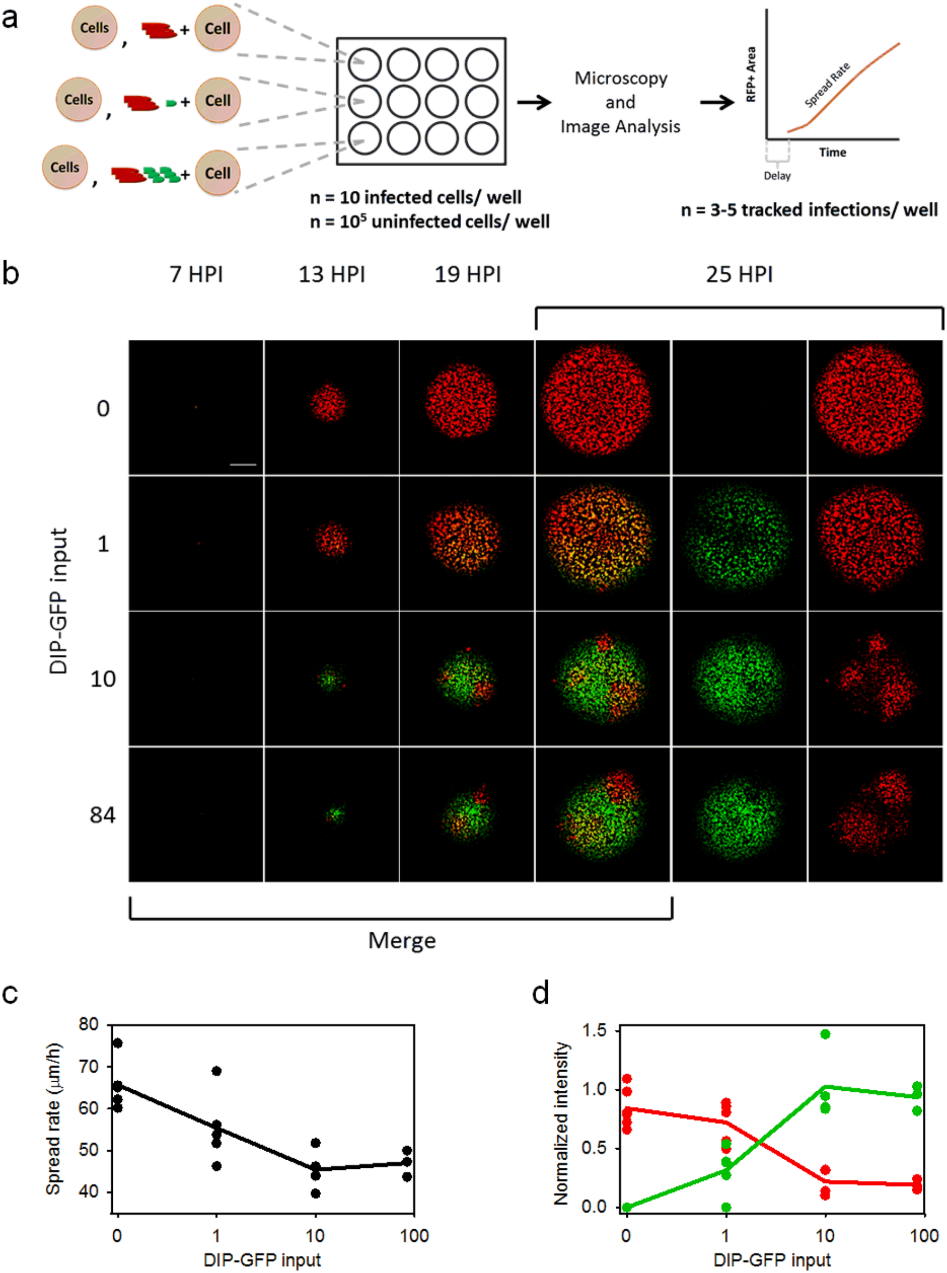
Co-propagation of infectious and defective virus. (a) Experimental setup. Cells were infected with a high MOI (30) of infectious virus and varying amounts of DIP-GFP(0-84). These infected cells were mixed with a large excess of uninfected cells, plated, and the spread progress of infectious and defective virus was observed via time-lapse microscopy. (b) Representative spreading infections. The first four columns contain merged images with red showing infectious virus expression, green showing defective virus expression, and yellow showing areas of both infectious and defective virus expression. The last two columns separate the red and green expression from the 25 hours post infection (hpi) images. The scale bar is 0.5 mm. (c) Infectious virus spread rate (*μ*m/h) as a function of DIP-GFP input. Individual plaques shown as points, the average as the line. (d) The normalized intensity during the earliest detectable spread, near plaque centers, for infectious (red points and line) and defective (green points and line) virus versus DIP-GFP input.

In principle, the interplay between infectious viruses and defective sub-viral particles in nature could play out over many rounds, with virus-infected cells and tissues providing a substrate for the co-production of defective particles. To test for this possibility a minority of cells infected with wild-type reporter virus, or co-infected with wild-type and DIP reporter viruses, were mixed with a large excess of healthy, susceptible cells. Plating them together produced a host-cell monolayer containing about ten infected cells per well (Fig 3A), and these cell-initiated infections were detected and tracked via time-lapse microscopy (Fig 3B). In the absence of DIPs, viral protein expression was detected about 7 hours post infection (hpi), and the infection appeared to spread at a uniform rate. When the plaque-initiating cells were supplemented with a low dose of DIP-GFP particles, infectious and defective viruses simultaneously spread, based on co-expression of both viral and DIP reporters during plaque expansion. These images provide the first direct evidence for the co-transmission of DIP and virus over multiple cycles of infection.

### Rates of infection spread depend on initiating DIP dose

Higher levels of DIP-GFP input to the initiating cell resulted in subsequent patchy spread, where some localized areas were dominated by DIP expression (green), and others were dominated by infectious virus expression (red). In all cases both the defective and infectious virus co-propagated well beyond the first infected cell to the surrounding cells in the monolayer. It should be emphasized here that the only source of virus or DIP in this spreading infection system is from the initial co-infected cell; no free virus or DIPs were added or present in the initial host cell monolayer.

To quantify the observed spread phenotypes, we first measured the area equivalent radius (AER) for total RFP-positive area above the detection threshold for all individual plaques (S3A Fig). The spread rate (average slope of AER vs time) and spread delay (time between infection initiation and fluorescence detection) were also determined (Fig 3 and S3B Fig). In the absence of DIPs, viral reporter gene expression was detectable at 7h post-infection, and the expression front spread with a uniform average rate of 65 *μ*m/h. In the presence of DIPs the delay was increased and the spread rate decreased as DIP-GFP input level changed from 1 to 10. Increasing DIP-GFP inputs from 10 to 84 had no detectable effects on the delay or spread rate. The impact of DIP-GFP dose on spread kinetics was mirrored in the effects on the RFP fluorescence observed near the plaque center (Fig 3D). Maximum integrated RFP and GFP intensities were measured within 120 *μ*m of the plaque center, an area that approximately accounts for the first two rounds of virus replication. There was a clear trade-off between infectious and defective virus yields around the plaque center, where the plaques that expressed the highest levels of defective virus protein (GFP) also expressed the lowest levels of infectious virus protein (RFP) (S3C Fig).

### Spatial distributions of virus replication depend on initial DIP dose

**Fig 4:**
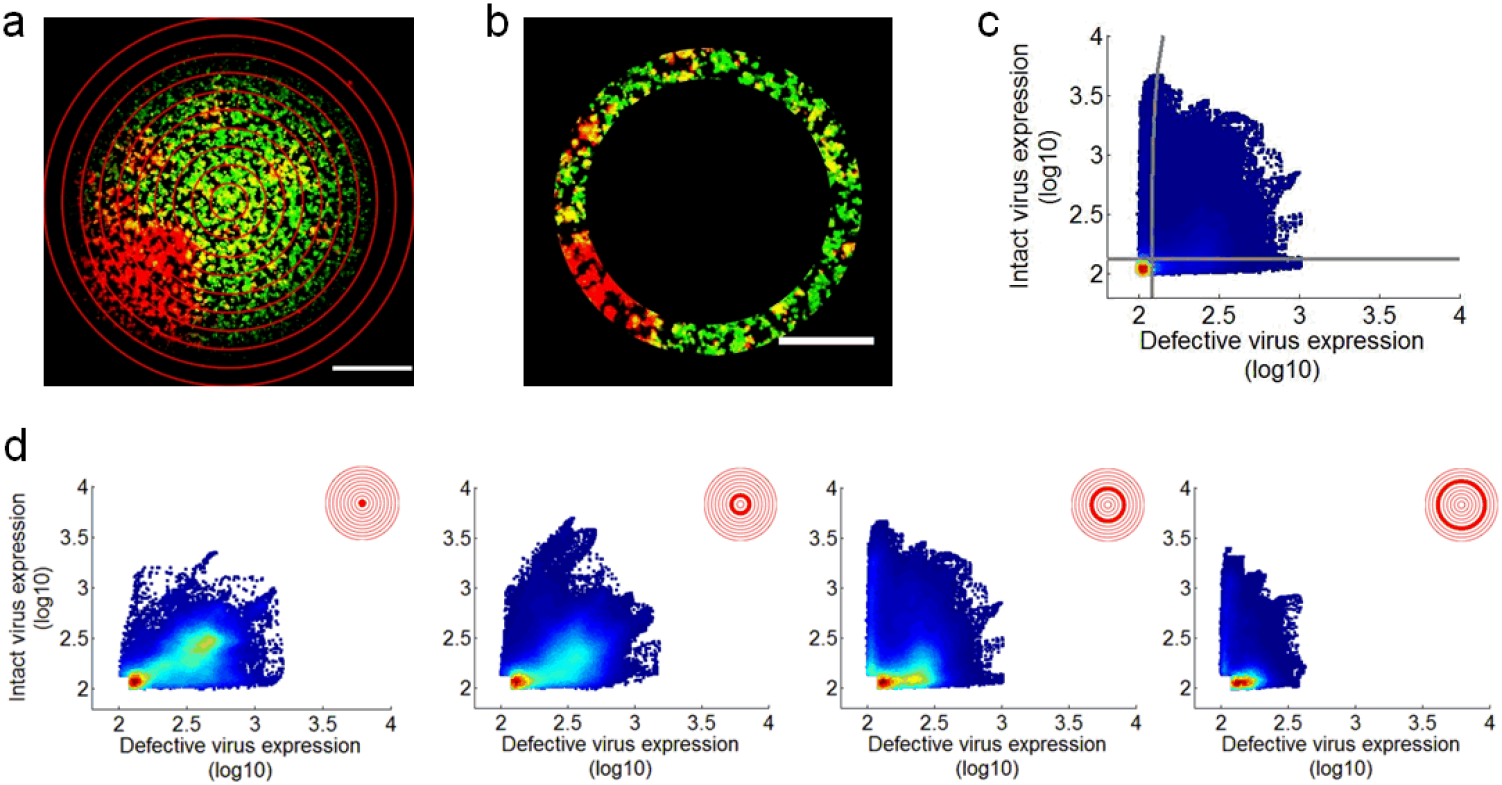
Spatial analysis of defective and infectious virus spread. (a) Example image (DIP-GFP input = 10) of a plaque maximum intensity projection with concentric rings overlayed, where the center is the plaque origin and each ring has a width of 100 pixels (116 *μ*m). (b) Example ring (r = 5) from the maximum intensity projection shown in (a). (c) The infectious virus (RFP) expression versus defective virus (GFP) expression for all pixels in (b). The color corresponds to point density with red being the highest and blue the lowest. The gray lines gate positive and negative populations. (d) The infectious virus (RFP) expression versus defective virus (GFP) expression for selected rings of the plaque; here, all pixel intensities that fall below both reporter thresholds have been excluded. For each ring, the contribution of each virus sub-population (e.g., infectious, defective or both) is shown as a percentage of the total population.

**Fig 5:**
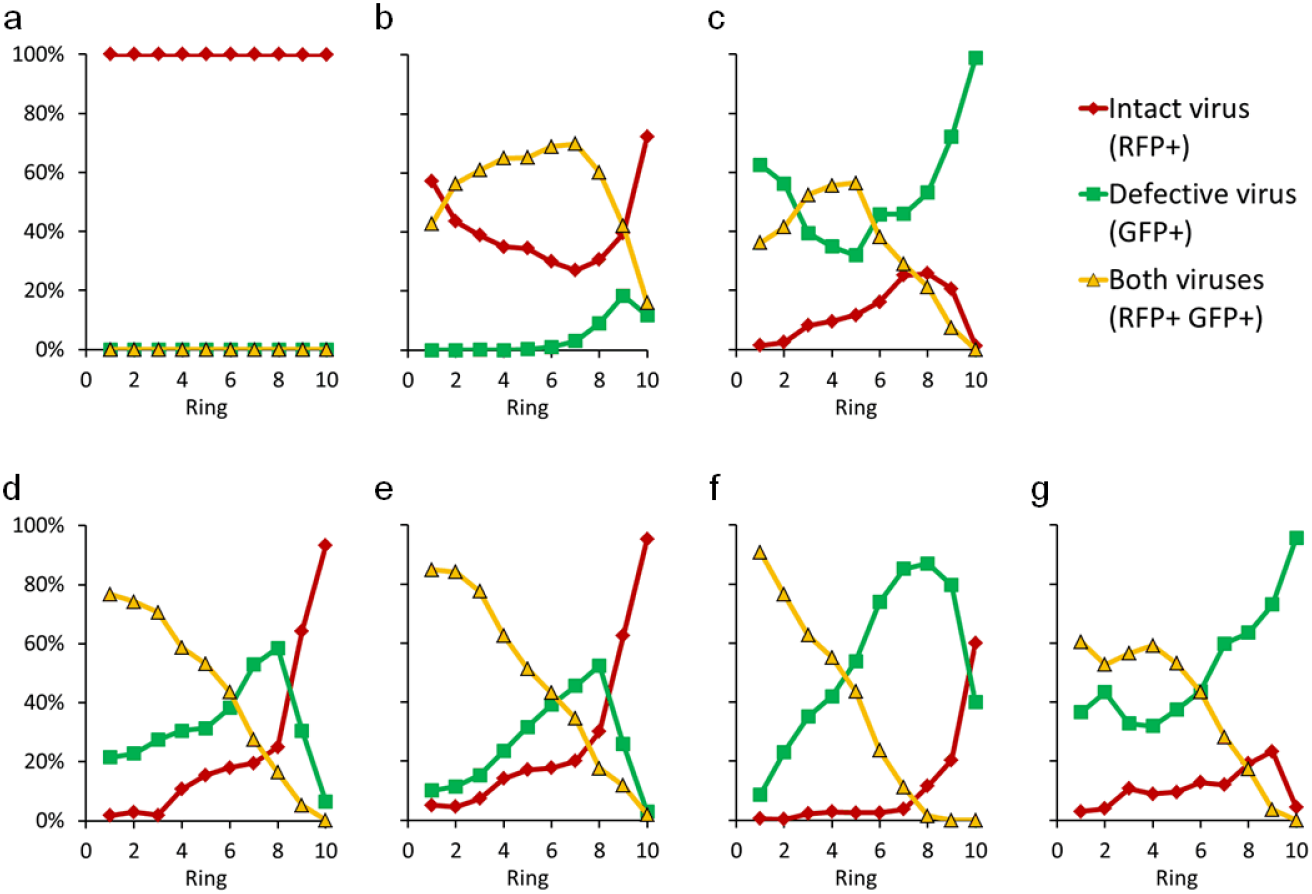
Spatial distributions of cells infected with infectious, defective and both viruses. The relative abundance of cells expressing reporter from infectious (RFP+), defective (GFP+), or both (RFP+, GFP+) viruses depends on distance from the plaque center (ring 1) as shown for representative plaques for DIP-GFP input levels (a) 0, (b) 1, and (c) 84 particles/cell. Plaques initiated with a DIP-GFP input of 10 particles/cell exhibited diverse patterns (d-g). Here, (d) shows the analysis for the plaque shown in Fig 4.

In spreading co-infections viral and DIP gene expression varied depending on time and location. To quantify these effects pixel-level intensities were analyzed along concentric rings of expanding infection, originating from the center of each plaque (Fig 4A). Here, the intensity of each pixel was set to its maximum value drawn from the full duration of plaque expansion; GFP and RFP maxima for each pixel were independently determined. The maximum projection was then segmented by concentric circles and the infectious virus expression (RFP) was plotted versus the defective virus expression (GFP) for every pixel in the ring (Fig 4B and 4C). Pixel values that fell below both the RFP and GFP thresholds were neglected, and the distribution of infectious and defective virus expression was tracked through space. For plaques initiated by cells co-infected with the two-reporters a majority of the pixels from the plaque center expressed both the infectious and defective virus reporter proteins (Fig 4D, left panel). However, across rings of ever larger plaque radii, distributions of maximal intensity often shifted between the two reporters; for example, in Fig 4d regions of two-reporter expression dropped from 77 percent to 28 percent, as regions of infectious and defective viral expression increased. Using this approach, multiple plaques were analyzed at each initial DIP dose. In the absence of any DIPs an initially infected cell expressed only RFP as the plaque expanded (Fig 5A). However, with an initial DIP-GFP dose of 1, an early dual-positive sub-population (ring 1) increased to a maximum and then declined, while an early infectious virus(RFP) sub-population declined to a minimum and then rebounded (Fig 5B). Plaques initiated by cells co-infected with an excess of reporter DIP over wild-type (84-to-30) were positive for GFP and RFP at the plaque center, but all such plaques were eventually highly enriched by GFP-only pixels with small patches of RFP-only areas (Fig 5C). Plaques initiated with an intermediate DIP-GFP input of 10 particles per cell had variable outcomes, with eventual enrichment by either GFP or RFP (Fig 5D through 5G). These results highlight the sensitivity of the population dynamics to the initial co-infection condition.

## Discussion

It has long been known that defective interfering particles can exploit the growth and spread of viable virus infections for their own reproduction. Interference of infectious virus growth *in vitro* has been inferred from highly variable yields of virus titer across fixed-volume serial-transfer cultures [27, 41], by measuring virus production from cells co-infected with viable virus and controlled dilutions of highly inhibitory stocks [42], or by quantifying the effects of fixed-MOI serial-transfers on viable virus production [27]. Less directly, interfering activity has been inferred by loss in pathogenicity or changes in recovery rates from viral infections in animal hosts [43, 44]. While single-cell measures of interference have highlighted the ability of DIPs to fully shut down virus progeny release from individual co-infected cells, measures from many individual cells and co-infected cell populations indicate DIPs cannot fully suppress virus production under any co-infection conditions [30, 37, 38]. Highly interfering stocks can significantly delay and reduce gene expression by infectious virus, but without complete shutdown of viable virus production from most co-infected cells [36]. Despite the demonstration of diverse interference measures, there have been few if any direct measures of DIP gene expression or interference function in co-infected host cells.

Here, by engineering DIPs one has the ability not only to create reporters of DIP intracellular development, but also to impact the expression of selected viral proteins and functions, and thereby potentially manipulate how DIP interferes with the development of infectious virus. By deleting the glycoprotein(G) from our reporter DIP genome, we created conditions for competition between the viral and DIP genomes for virally-expressed G protein, which is needed for infectious viral genome packaging and release. Future studies are planned to elucidate the mechanism of interference by quantifying how limited G-protein resources are distributed to competing viral or DIP genomes over the course of an infection cycle. For the present work, reporter DIPs were able to inhibit viral gene expression and production of viable viruses in a dose-dependent manner, reflecting similar behavior as non-reporter DIPs (Fig 1C and 1D). Moreover, higher input doses of DIP reporter particles in co-infected cells correlated with higher levels of DIP-associated gene expression (Fig 1D). Although gene expression levels varied widely with individual cells at a fixed level as well as across diverse levels of virus-DIP co-infection, overall trends were consistent with a competition for cellular resources at every input level. Specifically, higher inputs of reporter-DIP gene expression corresponded with lower levels of reporter-virus gene expression (Fig 2C). Moreover, by seeding co-infected cells into susceptible-cell populations we could observe how infection, triggered by progeny release from a single infected cell, exhibit diverse spatial-temporal patterns over multiple cycles of infection. The irregular or patchy spread of infectious reporter virus (RFP) previously observed for plaque growth initiated by DIP-virus co-infected cells was confirmed here, but more importantly, we could now directly observe gene expression and spatial spread of reporter DIPs, coupled with infectious virus infection spread. As with single-cycle co-infections, there were regions of greater DIP or infectious virus, where more robust expression of GFP or RFP corresponded with lower expression of RFP or GFP, respectively, consistent with a competition for limited host biosynthetic resources.

Asymmetry in the DIP-virus relationship was reflected in the appearance of RFP patches but no GFP patches. Infectious virus that diffuses to susceptible host cells may at any location initiate a productive infection that spreads, but that option is not open to DIPs. Nevertheless, DIPs here are found to very effectively co-infect with infectious virus over multiple cycles of spread, reducing the rate of co-infection spread relative to spread by virus alone (Fig 3C). In natural infections other factors may further inhibit the spread of virus alone or virus-DIP co-infections. Activation of innate immune signaling by infected cells can release cytokines, such as type-I interferons (IFNs), which reduce the susceptibility of natural hosts to further infection. Such signaling is not functional in the BHK host cells used here [45, 46]. Further, in other systems, notably Sendai and influenza A virus, DIPs have been found to potently activate innate immune signaling [47], and such activation may contribute to the development of protective immunity [48].

We did not determine genome sequences from any products of co-infection spread, so the possibility of mutation events by error-prone replication, recombination between reporter virus and reporter DIP, or deletion events cannot be definitively excluded. However, in the spatial patterns of co-infection spread no obvious products of mutation or selection were observed. One could imagine more robust virus growth or more robust DIP interference arising by loss of reporter genes from both engineered strains, respectively; however, the detection of at least one reporter within the bounds of each expanding co-infection plaque suggests such events were unlikely to have occurred.

Future work will seek to account for mechanisms of both DIP interference and innate immune activation in their effects on infection spread. Live-cell fluorescent reporting from IFN-sensitive promoters, which can be triggered by virus-infected cells and in nearby cells [34, 35], may be combined with reporters of viral and DIP gene expression to provide a more integrated and dynamic perspective. Mathematical models of the relevant interactions [25, 37, 49], guided by the experimental rich data, may enable extraction of further insights.

What role might DIPs play in the pathogenesis of viruses in nature? The questions and observations raised nearly half a century ago [50] remain open today, and they are increasingly relevant as more DIPs are isolated and characterized from natural and clinical sources. Could DIPs in nature or by human design be developed as anti-viral prophylaxis or therapies, as long proposed [43, 51, 52]? Diverse designs and approaches now being developed [53–55] may open new opportunities for protection against or treatment of persistent viral diseases.

## Materials and methods

### Cells and viruses

Baby hamster kidney (BHK-21 or BHK) cells provided by Isabel Novella (University of Toledo) were cultured at 37°C and 5% CO_2_ in Eagle’s minimum essential medium (MEM, CellGro) with 1% Glutamax I (Gibco) and 10% fetal bovine serum (FBS, Atlanta Biologicals). The same media with 2% FBS was used during all virus infections. The recombinant wild-type (WT, rVSV-WT) and WT-RFP (rVSV-WT-DsRedEx) virus strains used here have been characterized [34] and utilized in several studies [35, 36]; as engineered products of reverse genetics that have not been passaged at high MOI, they lack detectable DIPs. To produce DIP stocks, serial transfer infections of WT-RFP were carried out at fixed multiplicity of infection (MOI = 10), conditions that monotonically enrich for non-reporting DIP particles while driving WT-RFP levels below the limit of detection [36, 56]. The fluorescent, recombinant DIP strain (DIP-GFP) was produced by removing the glycoprotein gene from pVSV-FL(+) [57] by digestion with MluI and NheI (Fermentas) and inserting the eGFP gene via In Fusion cloning (Clontech). To recover infectious virus, BHK cells at 36°C were co-transfected with adapted plasmids pBS-N, pBS-P, and pBS-L for the expression of VSV genes N, P, and L [57, 58], and pMD2.G for the expression of the VSV glycoprotein (Didier Trono lab, Addgene), and infected with T7 expressing vaccinia virus [59]. The DIP-GFP master stock was purified via filtration with a 0.22 *μ*m Millex GV filter (Millipore) and stored at -80°C. The DIP-GFP working stock was prepared by infecting BHK cells transfected with pMD2.G. The particle concentrations of the DIP and DIP-GFP stocks were determined via tunable resistive pulse sensing [60, 61]. The concentration determined by these particle counts were similar to those determined by qPCR [56].

### Yield reduction assay

The reduction in infectious, wild-type virus production after co-infection with natural or recombinant DIPs was determined using the yield reduction assay [26]. This assay utilized serial dilutions of DIP or DIP-GFP to infect BHK cells in 96-well plates. The DIPs were adsorbed for 30 minutes then the cells were infected with WT-RFP virus at an MOI of 30. After an additional 30 minutes, the unbound WT-RFP virus was removed, the cells were rinsed with PBS, and MEM with 2% FBS was added. At 24 hpi the cells were imaged on a Nikon TE Eclipse 300 inverted microscope, and the supernatant was collected and stored at -80°C. The number of infectious virus particles in the supernatant was determined by plaque assay, and the total particle counts, which includes infectious and non-infectious particles, were determined using tunable resistive pulse sensing via a qViro (IZON Science, Christchurch, NZ) [60].

### Solution-phase co-infections

The co-infection of BHK cells with wild-type and DIP VSV strains has been described previously [36, 40]. BHK cells were grown in T-75 flasks (BD Falcon) and dissociated via trypsinization. These cells were suspended in MEM with 2% FBS at a concentration of 10^5^ cells/mL. The cell suspension was mixed with VSV-WT-RFP (MOI 30) and VSV-DIP-GFP (MOI 0, 1, 10, or 84) and incubated on ice for 30 minutes to enable virus attachment while preventing entry into the cell. After the attachment period, the cell suspension was subjected to three rounds of centrifugation and washing, performed at 1,000 rpm (50xg) for 4 minutes at 4°C followed by resuspension in MEM with 2% FBS, to minimize carryover of unbound virus. These infected cells were used to observe intracellular protein expression and spreading virus infections as described below.

### Cell plating for tracking individual cells

After the final centrifugation, the infected cells were resuspended in MEM with 2% FBS and Hoechst 33342 (1:20,000) (Anaspec, Fremont, CA) at a concentration of 5 × 10^5^ cells/mL. One mL of this solution was added into wells of a 12-well plate with two wells per DIP-GFP MOI condition. The cells were incubated at 37°C and 5% CO_2_.

### Cell plating for tracking spreading infections

To monitor spreading virus infections, cells infected as described above were diluted to a final concentration of 10 infected cells/mL in a suspension of 5 × 10^5^ uninfected BHK cells/ mL. One mL of this solution was added per well of a 12-well plate with two wells per DIP-GFP MOI condition. The cells were incubated for one hour at 37°C and 5% CO_2_ and then the media was removed and replaced with a semi-solid noble agar (Difco) overlay (0.6% w/v) in MEM with 2% FBS.

### Live cell microscopy

Cells were imaged via a Nikon Eclipse Ti inverted microscope equipped with a QICAM Fast 1394 digital CCD camera (Q Imaging, Surrey, BC, Canada). The cells were maintained at 37°C, 5% CO_2_, and 85% relative humidity via a stage top incubator (In Vivo Scientific) and a custom outer chamber maintained at 37°C by an In Vivo Scientific temperature controller. Illumination was provided by a Spectra X light engine (Lumencor), and the experiment was controlled with NIS Elements (Nikon).

Imaging for individual cell tracking was performed with a Nikon Plan Apo 10x, 0.45 NA objective lens beginning 1.5 hpi and continued every 10 minutes for 25 hours. The imaging for the spreading infections was performed with a Nikon Plan Apo 4x, 0.20 NA objective lens beginning 3 hpi and continued every 2 hours for 27 hours.

### Individual cell image processing and quantification

Image databasing and analysis was performed with JEX, an open-source application for high-throughput batch image processing [35]. Flat-field correction and background subtraction were applied to all fluorescent images. Individual cells were identified by finding local maxima in the images of the nuclear stain, and these were tracked through time via a nearest-neighbor approach. The RFP and GFP intensities were determined for a circle (diameter = 10 pixels) around each local maximum.

Several filters were applied to the RFP and GFP intensity data to ensure cell tracking fidelity using MATLAB R2013a (MathWorks, Natick, MA). For all cells, the RFP and GFP yields were calculated as the average of the 5 highest RFP or GFP intensities recorded through time [36]. The limit of detection (LOD, average plus four standard deviations of background intensity) was determined from a mock infected condition. If a cell’s yield was below the LOD in RFP or GFP, it was counted as a nonproducer for that fluorescence channel, and kinetic parameter analysis was not performed for the nonproducing channel. To account for cell fusion and mistakes in nuclear cell tracking, cells above the LOD with large fluorescence discontinuities (change equal to or greater than one quarter of the fluorescence yield within a 40 minute period) were discarded. Finally, cells with a continuous track of at least 7 hours were selected for kinetic parameter analysis.

The RFP and GFP kinetic parameters determined for each individual cell were latent time, rise time, and rise rate [36]. The latent time, which was also the beginning of the rise time, was defined as the first point in a continuous 1.5 hour period of fluorescence intensity above the LOD. The end of the rise time was recorded when the fluorescence intensity reached 85% of the final yield. Finally, the rise rate was calculated for the first 1.5 hours of the GFP or RFP rise time by fitting to an exponential function (*yield* = *A* * *e*^(rise rate*time)^) with a weight based on noise level [62].

### Spreading infection image processing and quantification

The images of spreading viral infections were databased and processed using JEX [35]. After flat field correction and background subtraction, the plaque centers were found from the earliest fluorescence detection and individual plaques were cropped using ImageJ [63]. The plaque area was determined for each plaque through time by approximating the RFP-positive area (threshold = average plus four standard deviations of background intensity) to a circle and recording the radius (area equivalent radius, AER). The spread rate was determined by calculating the slope of the linear approximation of AER over time, and the spread delay was the time between viral infection and fluorescence detection above the LOD.

### Plaque spatial analysis

To examine the plaque spatial features a maximum intensity projection was used. The intensity value of each pixel in the maximum projection was set equal to the maximum value observed through 25 hours post infection (hpi). The GFP and RFP maxima for each pixel were determined independently. The maximum projection was segmented into 10 concentric circles with the centers at the plaque origin and a radius that increased by 100 pixels (116 *μ*m) from one ring to the next. The pixel intensities from each ring were plotted with dscatter in Matlab R2013a [64, 65].

The RFP and GFP pixel values were gated into double negative, single positive, and double positive populations. The RFP and GFP threshold values were determined to be 133.2 and 120.6 respectively. These values are equal to the average plus four standard deviations of the background intensity from mock infected cells. To calculate the DsRedEx into the GFP channel compensation value, the no DIP condition was used (0.21%). There was no detectable spillover of eGFP into the RFP channel. Based on the threshold and compensation values the RFP gate was set at 133.2, and the GFP gate was set at 120.6 + 0.0021 × *RFPvalue*. The double-negative pixels below both the RFP and GFP gates were discarded and population distributions of double-positive, RFP-positive, and GFP-positive pixels were followed through space.

## Supporting information

S1 Fig DIP and intact virus single-cell kinetic parameters. Intact and defective virus expression kinetics in single cells after different doses of defective virus.
(TIF)

S2 Fig Correlation between single-cell intact (RFP) and defective (GFP) virus expression kinetic parameters. For all single cells with detectable RFP and GFP expression, the kinetic parameters (a) latent time, (b) rise time, and (c) rise rate are plotted.
(TIF)

S3 Fig DIP and intact virus spread features. (a) The area equivalent radius of total RFP positive area is plotted over time for all individual plaques. (b) The effect of DIP-GFP input on the plaque delay time. Each point is an individual plaque and the line passes through the average for each DIP-GFP input level. Multiple plaques have the same delay value so the points overlap. (c) Anticorrelation between defective and intact virus expression. For each individual plaque the defective virus (GFP) intensity is plotted versus the intact virus (RFP) intensity.
(TIF)
