# Supplementary figures and images for: Inhibition of infection spread by co-transmitted defective interfering particles
Source: PLoS One. 2017 Sep 15;12(9):e0184029. doi: 10.1371/journal.pone.0184029 (PMC5600374; doi:10.1371/journal.pone.0184029)

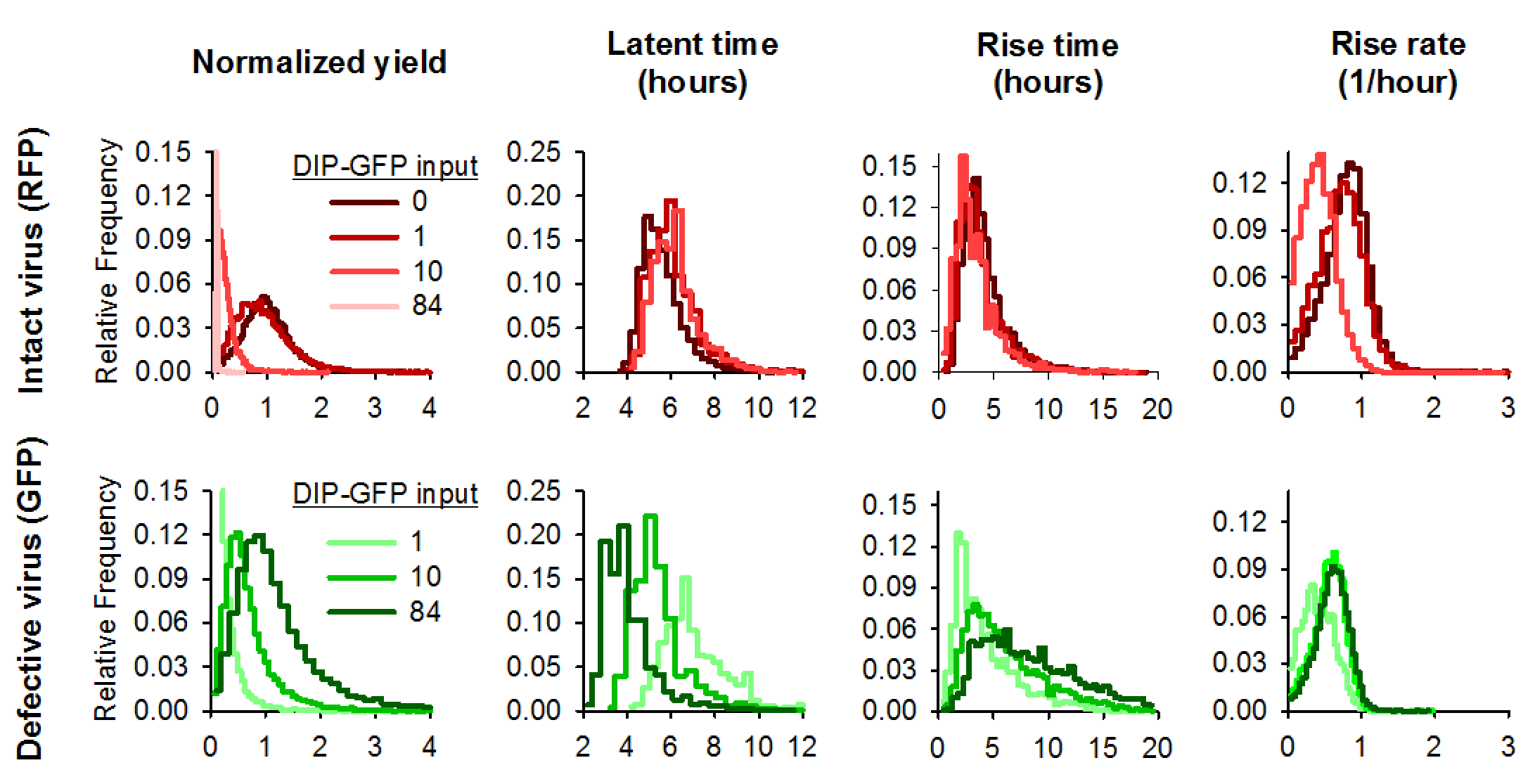

Supplement: S1 Fig — Intact and defective virus expression kinetics in single cells after different doses of defective virus. (TIF) [file pone.0184029.s001.tif]
